# A Rare Case of Intestinal Low-Grade Endometrial Stromal Sarcoma With Glandular Differentiation and Associated Endometriosis

**DOI:** 10.7759/cureus.14801

**Published:** 2021-05-02

**Authors:** Azka Anees, Khurram Siddique, Hisham Abouzeid, Sami Titi

**Affiliations:** 1 Histopathology, Northern Care Alliance National Health Service (NHS) Trust, Oldham, GBR; 2 Colorectal Surgery, Northern Care Alliance National Health Service (NHS) Trust, Oldham, GBR; 3 Obstetrics and Gynaecology, Northern Care Alliance National Health Service (NHS) Trust, Oldham, GBR

**Keywords:** endometrial stromal sarcoma, endometriosis, extra-uterine stromal sarcoma, liver metastasis, small intestine

## Abstract

Endometrial stromal sarcoma is a rare tumour. It is even rarer to find it arising in the background of endometriosis in an extrauterine location. This case report describes a case of missed diagnosis of intestinal extra-uterine endometrial stromal sarcoma associated with endometriosis, and the subsequent presentation with distant metastases. The potential pitfalls are highlighted and differential diagnoses are discussed.

## Introduction

Endometriosis is quite common in the reproductive age group with a reported incidence of 10% [1]. Of all these cases, intestinal involvement is seen in 3% to 37% [2,3]. Malignant transformation in endometriosis has been well-documented but is rare, only occurring in 0.7%-0.1% of cases [2]. It is believed that any type of tumour found in the endometrium can also arise from endometriosis [4], the most common ones being endometrioid and clear-cell type carcinomas [5]. It is exceedingly rare to see an extrauterine endometrial stromal sarcoma (ESS) arising from endometriosis [4].

ESS is characterized by cells that resemble proliferative phase endometrial stromal cells. It usually originates from the uterine corpus, but may be seen in extra-uterine locations [6]. ESS arising from endometriosis has been reported to be an indolent tumour with a very good prognosis. However, high-grade cases have a poorer prognosis with a mean survival of 53 months [7]. Even low-grade tumours can present with late recurrences up to three decades after diagnosis [8]. They can also rarely metastasize to other organs [9] and treatment becomes especially problematic in cases with disseminated disease.

We report a case of missed diagnosis of intestinal extra-uterine endometrial sarcoma associated with endometriosis, and subsequent presentation with distant metastases.

## Case presentation

A 46-year-old female patient was admitted to the hospital with abdominal pain and vomiting and had a CT scan of the pelvis which showed a complex adnexal mass and large mixed density mesenteric lesion in association with small bowel loops (20 x 15 cm). The patient underwent laparotomy where the mesenteric tumour and small bowel loop were excised and sent for histological examination along with a clinical diagnosis of possible mesenteric lymphangioma or endometriotic cyst. There was no definite adnexal mass found during surgery and liver and rest of the bowel were unremarkable.

**Figure 1:**
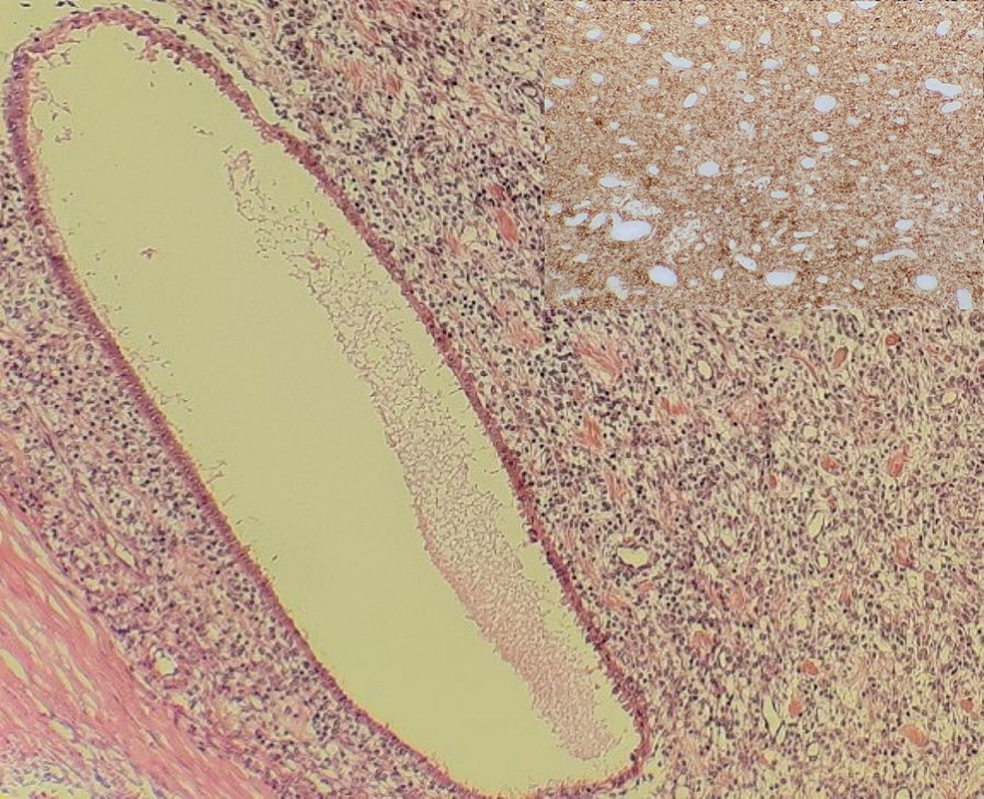
(H&E, 10X): Microscopic appearance of mesenteric mass in small bowel showing monotonous stroma and endometrial type glands. Inset: (CD10, 10X): CD10 showed diffuse positive staining in stromal cells of small bowel mesenteric mass.

The histological examination of the mesenteric mass showed benign-looking endometrial type glands surrounded by monotonous endometrial stromal cells which revealed no obvious cytological atypia (Figure 1). Immunohistochemistry for CD10 highlighted the endometrial stromal cells and an initial diagnosis of endometriosis was made based on the overall appearance (Figure 1, inset).

The patient recovered well after surgery and was discharged in a satisfactory condition. However, after 14 months from the first admission, she presented to the accident and emergency department (A&E) with abdominal pain.

CT scan of the chest, abdomen, and pelvis was done which showed cystic solid liver lesions, pulmonary micronodules, omental infiltration, and soft tissue deposits. A suspicion of metastatic malignancy was raised at this point and correlation with the previous histology examination of the mesenteric mass was advised.

**Figure 2:**
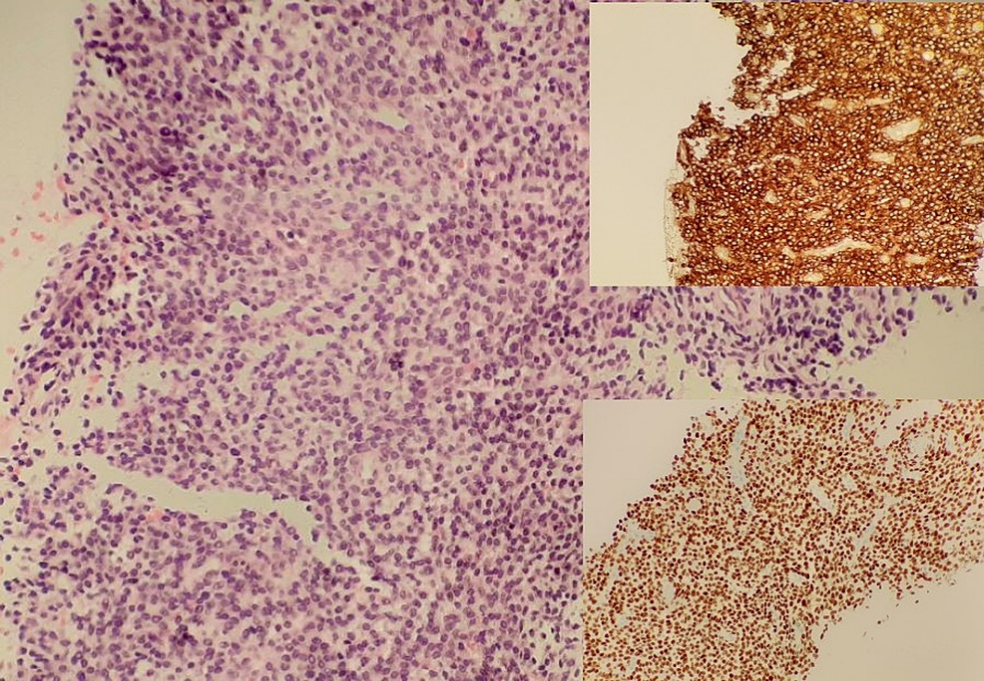
(H&E, 20X): Microscopic appearance of liver biopsy which shows complete replacement by tumour cells. Inset (top): Diffuse strong positivity for CD10 in liver biopsy. Inset (bottom): Diffuse strong positivity for ER in liver biopsy.

An ultrasound-guided liver biopsy was performed and sent for histology. The liver biopsy showed complete replacement by spindle cells with a positive expression for CD10 and ER (Figure 2, insets). No normal liver parenchyma was identified. The stains for MNF116, D2-40, calretinin, AE1-3, Desmin, S100, CD34, CK7, CK20, Melan A, CD117, p53, and beta-catenin were negative.

It was noted that the changes were of similar appearance to the stromal changes in the small bowel resection received earlier. A possible diagnosis of a stromal component in endometriosis or ESS was considered. After external review, it was agreed that the liver biopsy contained ESS and the appearances were in keeping with those of metastatic ESS in which the tumour is of uniform “low grade” appearance.

**Figure 3:**
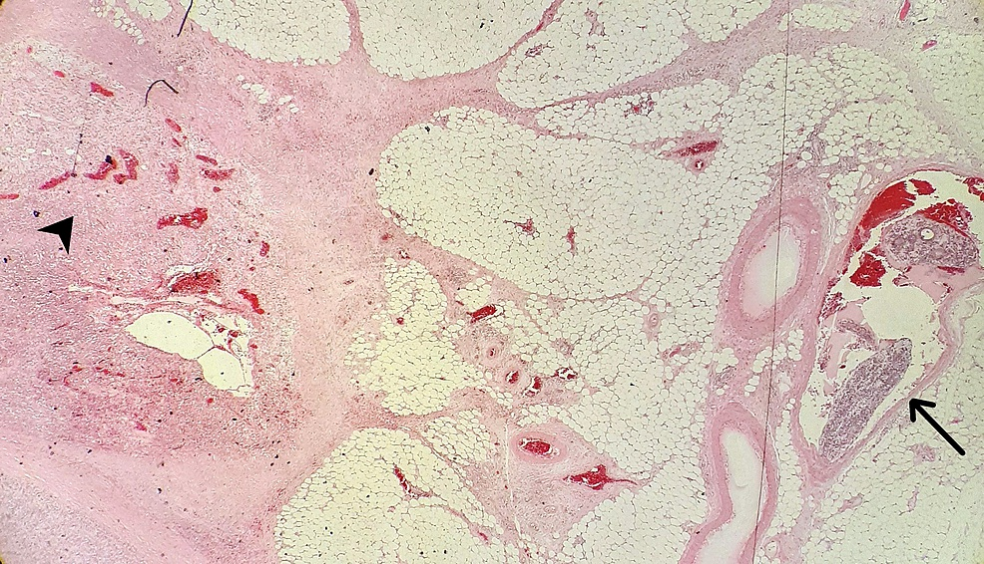
(H&E, 10 X): Vascular thrombi of tumour in section from small bowel mesenteric mass. The endometrial stromal sarcoma is seen on the left side of the image (arrow head) with a large vessel containing tumour thrombi on the right (arrow).

This led to a diagnostic review of the small bowel resection reported previously. It was agreed that it contained definite endometriosis. In addition, there were features of associated ESS. There were admixed endometrial glands within the sarcoma which were presumed to represent endometrioid differentiation within stromal sarcoma. Mitotic activity was infrequent (<5 mitoses per 10HPF). A focus of lymphovascular invasion was also noted in the review (Figure 3).

A final diagnosis of low-grade ESS with relatively prominent endometrioid glandular differentiation and associated endometriosis was given.

## Discussion

This case is being highlighted to discuss its complexity and associated pitfalls. The case was discussed at a discrepancy meeting in the histopathology department and there was consensus that this case was challenging. None of the participants had come across such a case in their career. In addition to the rarity of the disease, incomplete clinical details on the request form (sent with specimen), without any suspicion of malignancy, from the surgeons contributed to the missed diagnosis. The presence of this information may have supported a diagnosis of malignancy at the time of the original pathology report. It was agreed that vigilance is required when dealing with extrauterine endometriosis and in particular when the stromal component shows exaggeration and prominent expansion.

ESS is a rare tumour with about 80 cases reported so far [10,11]. A case series from M D Anderson Cancer Centre, Texas, reports the largest number of such cases. This case series described the most common sites of ESS as the abdominal peritoneum and bowel wall. It was reported that half of these sarcoma cases were associated with endometriosis.

The development of malignancy in endometriosis was studied quite early. In 1925, Sampson [12] reported the first case series of malignancy arising from endometriosis and recommended three criteria for its diagnosis: (1) examples of endometriosis in close proximity to the tumour; (2) no other primary site of malignancy, and (3) histological appearance compatible with the origin from endometriosis. These are helpful criteria and easily applicable in routine practice.

As seen from the case above, the diagnosis of low-grade ESS with prominent glandular differentiation is difficult, especially when the primary presentation is in the intestine. It can be confused with many other lesions, some of which will be discussed below.

The main diagnostic difficulty in our case was the differentiation between endometriosis and ESS with extensive glandular differentiation. This is understandably difficult and some light is shed on this topic by a research article by McCluggage et al. [13]. This article studies the unusual occurrence of ESSs with extensive endometrioid glandular differentiation and highlights the heterologous morphology of these tumours, with some cases showing glands throughout the neoplasm, and others with areas of typical ESS without glands. Glands were seen only in the recurrent tumour in one case. The article also highlights the increased chances of initial misdiagnosis and delayed diagnosis causing patient harm, much like our case. Few characteristic morphologic features have been defined which can be helpful in the diagnosis of this challenging entity. These include invasive “tongues” of tumour at the periphery of the neoplasm, short fascicles or sheets of monotonous plump spindle cells, and prominent arterioles [14].

Another important differential for our case was stromal endometriosis, which is known to simulate a sarcoma [15]. Stromal endometriosis is a well-circumscribed lesion composed of endometrial stroma type cells with absent or very scant endometrial glands. The predominance of stroma and lack of endometrial glands can lead to confusion with several neoplasms, particularly low-grade ESS.

Aggressive endometriosis is another entity commonly confused with extrauterine ESSs [16]. This differentiation is particularly problematic as aggressive endometriosis is known to disseminate to different organs. Intravascular intrusion, perineural space invasion, and involvement of lymph nodes have also been documented in these cases [17]. A useful histological clue to indicate a diagnosis of aggressive endometriosis over ESS is the presence of endometrial stroma with glands that show normal cyclical changes according to the menstrual cycle. There should be no stromal overgrowth and p53 overexpression should be demonstrable on immunohistochemistry in cases of aggressive endometriosis [16].

Other differential diagnoses of primary extrauterine stromal sarcoma in the intestine include gastrointestinal stromal tumour (GIST) and Mullerian adenosarcoma with sarcomatous overgrowth. GIST is reported as the most prevalent stromal tumour in the intestine. It is important to differentiate ESS from GIST as the management route for both of these tumours is completely different. GISTs are well-circumscribed and have pushing borders in contrast to ESS which is invasive. Positive immunohistochemistry with c-kit is usually confirmatory for a diagnosis of GIST.

A less common condition that can be included in the differential diagnosis is Mullerian adenosarcoma with sarcomatous overgrowth. The main histological clue to this diagnosis is the presence of different kinds of Mullerian epithelium. Other findings are periglandular cellular stromal condensation and extension of cellular stroma into the lumen of glands.

## Conclusions

The diagnosis of intestinal low-grade ESS with endometrioid glandular differentiation and associated endometriosis is difficult with a high likelihood of misdiagnosis and delayed diagnosis. A careful morphological and immunohistochemical examination is the key to the correct diagnosis.
